# Dual orexin receptor antagonists for insomnia in youth with neurodevelopmental disorders: a case series and review

**DOI:** 10.1007/s00787-021-01883-7

**Published:** 2021-10-05

**Authors:** Aaron D. Besterman, Shafali S. Jeste

**Affiliations:** 1 Department of Psychiatry, University of California San Diego, San Diego, CA USA; 2 Rady Children’s Hospital of San Diego, 3020 Children’s Way, San Diego, CA 92123 USA; 3 Rady Children’s Institute for Genomic Medicine, San Diego, CA USA; 4 Semel Institute for Neuroscience and Human Behavior, University of California Los Angles, Los Angeles, CA USA; 5 Division of Child Neurology, Department of Pediatrics, UCLA David Geffen School of Medicine, University of California Los Angeles, Los Angeles, CA USA; 6 Departments of Neurology and Pediatrics, Keck School of Medicine, University of Southern California, Los Angeles, CA USA; 7 Division of Neurology and Neurological Institute, Children’s Hospital Los Angeles, Los Angeles, CA USA

**Keywords:** Insomnia, Neurodevelopmental disorders, Dual orexin receptor antagonists, Sleep disorders

## Abstract

Insomnia is a common, impairing, and difficult-to-treat comorbidity in children with neurodevelopmental disorders (NDDs). Behavioral interventions can be challenging because of developmental and behavioral features that interfere with treatment. Medication management also can be difficult due to a high burden of side effects, a high rate of paradoxical responses, and frequent treatment resistance. Therefore, new treatment options for insomnia in children with NDDs are needed. Dual orexin receptor antagonists (DORAs) are a relatively new class of pharmacotherapeutics that induce sleep by inhibiting the orexin signaling pathway. To date, there is little safety or efficacy data on the use of DORAs in children with NDDs. We present four patients with NDDs and insomnia that we treated with the DORA, suvorexant. We found that patients had a wide range of responses, with one patient displaying a robust improvement in sleep onset and maintenance, while another had significant improvement in insomnia symptoms on combination therapy with trazodone. Our final two patients had mild or no benefit from suvorexant therapy. Further research is necessary to establish the safety and efficacy of DORAs in this population and to identify predictive factors, such as specific neurogenetic diagnoses or clinical features, of a positive treatment response.

## Introduction

Sleep disorders are common in patients with neurodevelopmental disorders (NDDs), such as global developmental delay (GDD), autism spectrum disorders (ASD) and intellectual disability (ID) [1]. While prevalence estimates vary depending on how sleep disorders are defined and on the specific group of patients studied, sleep problems in patients with NDDs are linked to negative functional outcomes, such as daytime sleepiness, worsening cognitive function, irritability, and challenging behavior [1]. A diagnosis of insomnia requires (1) a report of sleep initiation or maintenance problems, (2) adequate opportunity and circumstances to sleep, and (3) daytime consequences [2]. Behavioral interventions are first-line treatments for insomnia, such as improving the child’s sleep environment or creating a more regular bedtime routine [3, 4]. However, behavioral comorbidities associated with NDDs, such as hyperactivity, irritability or insistence on sameness, can limit the effectiveness of behavioral interventions making pharmacotherapies necessary.

Melatonin has the most robust safety and efficacy data for insomnia in patients with NDDs [5], but there are additional pharmacotherapy options (Table 1). Patients with NDDs tend to have a high burden of side effects at low doses, such as behavioral activation with benzodiazepines. Additionally, there are high rates of treatment resistance and a pressing need for new treatment options. Dual orexin receptor antagonists (DORAs) are a relatively new class of pharmaceuticals approved in the United States, Japan and Australia (but not yet Europe) [6] for the treatment of adult insomnia [7]. They reduce orexin signaling by blocking both orexin receptors 1 and 2, thereby inhibiting arousal and inducing a state of sleep [7]. There are potential benefits of DORAs over traditional insomnia pharmacotherapeutics, such as promoting rapid eye movement (REM) sleep in addition to non-rapid eye movement (NREM) sleep in adults [8]. There is no evidence of habit and tolerance formation as with sedative hypnotics, benzodiazepines and antihistamines, or metabolic side effects as with atypical antipsychotics [7]. Excess orexin signaling with decreased REM sleep has been observed in an animal model of one NDD, Tuberous Sclerosis Complex, which was rescued by DORA treatment [9]. However, there is little clinical safety and efficacy data on the use of DORAs in the pediatric population [10] or in NDDs specifically [11, 12] (Table 1).

**Table 1:** Previous reports on the use of suvorexant in youth with sleep disorders

Study	*N* (Age range)	Suvorexant Dose	Sleep Disorder	Psychiatric and/or Neurodevelopmental Disorder(s)	Outcome	Side Effects
Kawabe et al*.* [10]	30 (10–20)	20 mg	Insomnia	Mix of comorbid psychiatric disorders; seven patients with ASD	No significant difference in sleep between those who stayed on suvorexant and those who discontinued it	Two patients with nightmares
Oka et al*.* [11]	3 (13–19)	15-20 mg	Circadian rhythm sleep–wake disorders	Two patients with ASD	One fell asleep two hours earlier, one had sleep phase advance two hours, one had a fixed nocturnal sleep schedule	None reported
Iwata et al. [12]	1 (23)	15 mg	Non-24-h sleep–wake disorder	ASD	Sleep rhythm restoration*	None reported
Prieto et al*.* [14]	1 (16)	10 mg	Insomnia	Bipolar disorder	Sleep duration improved from 3–4 to 6–7 h per night, sleep quality improved, and the patient reported more refreshing sleep	None reported

*Treatment in combination with ramelteon, *ASD* Autism spectrum disorder, *mg* milligrams, *N* number of patients

## Methods

We share our experience using one DORA, suvorexant, in four patients with NDDs with clinically diagnosed insomnia who failed all other standard treatments. All patients and/or guardians provided informed consent for publication of de-identified data (UCLA IRB 14-001908).

## Results

Patient 1 was a 9-year-old female with Kleefstra Syndrome (Online Mendelian Inheritance in Man: 610253), ID, adrenal insufficiency, hypogammaglobulinemia, asthma, anismus, sleep maintenance insomnia, and obstructive sleep apnea. Her failed medication trials for insomnia included melatonin, trazodone, gabapentin, clonidine, guanfacine, cyproheptadine, and amitriptyline. We first started mirtazapine, which had little impact on her sleep and caused significant weight gain. We then started suvorexant 5 mg nightly, which reduced the number of nighttime waking episodes. Its benefit for sleep maintenance dissipated over the course of several weeks, even on doses up to 15 mg nightly, which was reached by titrating up by 5 mg approximately once a month for 2 months. Based on a previous case series in Kleefstra Syndrome [13], we switched her to quetiapine 12.5 mg nightly, with marked improvement in sleep maintenance.

Patient 2 was a 14-year-old male with asthma, ID, ASD, agitation and insomnia with sleep initiation insomnia. He had fragile X and chromosomal microarray testing at an outside institution, but the results were not available. He would stay awake until 5 am and then fall asleep for a few hours, having to then wake up for school. He was tired and irritable all day but could not fall asleep at bedtime. On intake, he was taking lorazepam 2 mg nightly for anxiety and insomnia. He had previous failed trials of melatonin (agitation), fluoxetine and sertraline (mood disturbance, agitation, gastrointestinal upset). We started him on clonidine 0.1 mg nightly and switched his lorazepam to clonazepam 0.25 mg twice daily. His insomnia persisted and agitation worsened with clonidine, so it was stopped. He was titrated up to 75 mg nightly of trazodone, with transient improvement in sleep initiation but ongoing difficulties with sleep maintenance. Suvorexant 5 mg was added and titrated up to 15 mg nightly by increasing the dose by 5 mg per month over 2 months, with significant improvements in both sleep initiation and maintenance that has been sustained for over a year now without emergence of additional side effects.

Patient 3 was a 28-year-old with ID, ASD, complex partial seizure disorder, bipolar disorder, aggressive behavior, obesity, and sleep initiation and maintenance insomnia. He was referred to medical genetics, but the outcome of that referral is undetermined. He had been tried on many psychotropic medications for behavior, epilepsy, and insomnia (full list unknown). He was stabilized on oxcarbazepine 300 mg twice daily, lamotrigine 300 mg twice daily, aripiprazole 10 mg nightly, and clonidine 0.1 mg nightly. His insomnia persisted, taking hours to fall asleep, and waking many times throughout the night. He was started on suvorexant 5 mg and titrated up to 15 mg over the course of several months. His sleep improved dramatically, falling asleep within 90 min and remaining asleep for 6–8 h per night. This was the best sleep he had “in years”, per his mother. He subsequently lost more than 100 pounds due to increased daytime energy, his mood improved substantially, and he was able to attend an adult day program.

Patient 4 was a 13-year-old female with ASD, ID, epilepsy, obsessive–compulsive disorder, anxiety and insomnia. She had a normal chromosomal microarray, negative fragile X and *MECP2* testing, and an autism gene panel revealed a maternally inherited (non-pathogenic) variant in *NSD1*. She had been tried on multiple psychotropics without significant benefit, including risperidone, clonidine, valproic acid, memantine, aripiprazole, and sertraline. On intake, she was on fluoxetine 5 mg daily, quetiapine 75 mg nightly and melatonin 6 mg nightly. She continued to have poor sleep maintenance, so was trialed on suvorexant 5 mg nightly without improvement or major side effects, so it was discontinued.

## Discussion

We reported one male and three female patients with NDDs and insomnia who ranged in age from early childhood to early adulthood. They were all diagnosed with ID and all but one with ASD. They had a range of medical, neurological and psychiatric co-morbidities and one had a genetic diagnosis, Kleefstra Syndrome. Our patients had a range of responses to suvorexant, from dramatic and sustained improvement in sleep and quality of life (patient 3), to little to no benefit (patients 1 and 4). No major side effects were observed. In comparison, in an open-label study of suvorexant in thirty adolescents, no significant difference in sleep quality was seen at 6 months between the 17 patients on suvorexant compared to the 8 who completed the study but discontinued suvorexant [10]. The only side effect reported were nightmares in two patients. Seven of the patients had ASD, but no subgroup analysis was reported [10]. A case series of three adolescents with circadian rhythm sleep–wake disorders, two with ASD, reported subjective improvements in sleep schedules [11] and one youth with insomnia and bipolar disorder had improved sleep duration on 10 mg of suvorexant [14]. In clinical trials of neurotypical adults with insomnia, suvorexant was associated with significant improvements in subjective time-to-sleep onset, total sleep time, and quality of sleep [15]. Participants reported elevated rates of somnolence, fatigue, abnormal dreams, and dry mouth [15].

These data suggest that suvorexant may be safe and effective for certain patients with NDDs, but additional research with prospective clinical trials in this specific population is necessary, especially in very young populations where the data are extremely sparse. Of note, the one patient of ours with a highly positive DORA response was a young adult (patient 3, age 28). Most trials in the general adult population used 20–40 mg [15], but we prescribed 5–15 mg out of caution, given the known sensitivity of patients with NDDs to psychotropic side effects. Additional caution is necessary with co-administration of CYP3A inhibitors, such as protease inhibitors or azole antifungals, as suvorexant is primarily eliminated through the CYP3A pathway [16]. Conversely, suvorexant may have reduced effectiveness in patients on CYP3A inducers, such as oxcarbazepine, carbamazepine, or phenytoin [16]. There are currently no biomarkers or clinical features predictive of DORA response, so clinical judgement must be used to select appropriate patients. Given established safety and effectiveness of other insomnia pharmacotherapeutics (Table 2), DORAs should only be considered for patients with NDDs who do not respond to or cannot tolerate all other established pharmacotherapeutics. There may be a genetic subpopulation of patients with NDDs who uniquely respond to suvorexant due to orexin signaling dysfunction, such as tuberous sclerosis complex [9], but this has yet to be confirmed. There is precedent for genetic diagnoses guiding sleep management. Patients with Smith–Magenis Syndrome have an inverted melatonin circadian rhythm that can be corrected through suppression of morning endogenous melatonin with beta blockers and evening supplementation with exogenous melatonin [17]. Thus, a gene-first approach may allow for the identification of DORA-responsive patients in the future.

**Table 2:** Pharmacotherapeutic options for insomnia in patients with neurodevelopmental disorders

Class of Medication	Example Medications	Potential benefits for patients with NDDs	Potential concerns for patients with NDDs
Exogenous Hormone	Melatonin	Available over the counter with robust safety and efficacy data in pediatric and NDD populations	No long-term data on impact of neurodevelopment
Melatonin Agonists	Ramelteon Tasimelteon	Relatively safe with few side effects, longer acting-than melatonin. Ideal for patients with non–24-h sleep–wake disorder	Relatively little data in pediatric and NDD populations
Alpha-agonists	Clonidine Guanfacine	Ideal for patients with comorbid ADHD, tics, or irritability	Generally well tolerated. Small risk of anticholinergic side effects and hypotension, dizziness and syncope
Antihistamines	Diphenhydramine Hydroxyzine	Ideal for brief, occasional insomnia. Diphenhydramine is available over the counter	Risk of paradoxical disinhibition, anticholinergic side effects, and development of tolerance
Non-benzodiazepine Sedative Hypnotics	Zolpidem Zaleplon Eszopiclone	Generally contraindicated in patients with NDDs because of high risk of paradoxical response	Very limited data in pediatric populations, risk of disinhibition, parasomnias and hallucinations
Benzodiazepines	Lorazepam Tenazepam Clonazepam	Ideal for patients with comorbid parasomnias or panic disorders	High risk of paradoxical responses, risk of developing tolerance, can be habit forming, takes time to taper safely
Tricyclic Antidepressants	Amitriptyline Doxepin	Ideal for patients with comorbid depression, anxiety, or functional gastrointestinal disorders	Limited data in pediatric and NDD populations. High risk of anticholinergic side effects, sexual dysfunction, increased suicidality in children, adolescents and young adults
Tetracyclic Antidepressants	Mirtazapine	Ideal for patients with comorbid depression, anxiety, or functional gastrointestinal disorders	May cause moderate weight gain, risk of mania with bipolar disorder, increased suicidality in children, adolescents and young adults
Atypical Antipsychotics	Quetiapine Olanzapine	Ideal for patients with comorbid agitation/aggression, psychosis, or bipolar disorder	Significant metabolic side effects, risk of extrapyramidal symptoms and lowering of seizure threshold
Gabapentinoids	Gabapentin Pregabalin	Ideal for patients with comorbid pain disorders, anxiety disorders, or epilepsy. Relatively safe	Limited safety and efficacy data in pediatric and NDD populations

*ADHD* attention-deficit hyperactivity disorder, *NDD* neurodevelopmental disorder

In conclusion, our four patients with NDDs and insomnia had variable but promising responses to treatment with suvorexant. The use of DORAs in patients with NDDs must be approached with caution given the lack of current safety and efficacy data in pediatric and NDD populations. Off-label use may be appropriate in cases when no established treatment works if caution is used and side effects are closely monitored, but this needs to be studied further. Specifically, clinicians should discuss the limitations to our current knowledge base about the effects of suvorexant on children with NDDs (as no rigorous clinical trials have been completed), the known side effects from studies of other populations (e.g., abnormal dreams, daytime drowsiness, dry mouth), the possibility of paradoxical or unknown side effects, and the importance of starting at low doses and titrating slowly to find the minimal effective dose. One limitation to our study was that our patients did not have specialized sleep evaluations that included polysomnography or actigraphy. Insomnia diagnoses were made based on clinical history, reflective of the common clinical situation most providers are faced with when treating sleep disorders in patients with NDDs. However, in future clinical studies to establish safety and efficacy and to identify predictors of DORA response in patients with NDDs, a more formalized sleep assessment will be beneficial to establish a definitive insomnia diagnosis and assure a more homogeneous study population.
